# Thermodynamic Insights and Conceptual Design of Skin-Sensitive Chitosan Coated Ceramide/PLGA Nanodrug for Regeneration of Stratum Corneum on Atopic Dermatitis

**DOI:** 10.1038/srep18089

**Published:** 2015-12-15

**Authors:** Sang-Myung Jung, Gwang Heum Yoon, Hoo Chul Lee, Moon Hee Jung, Sun Il Yu, Seung Ju Yeon, Seul Ki Min, Yeo Seon Kwon, Jin Ha Hwang, Hwa Sung Shin

**Affiliations:** 1 Department of Biological Engineering, Inha University, Incheon, 402-751, Korea

## Abstract

Atopic dermatitis (AD) is a complex skin disease primarily characterized by psoriasis of the stratum corneum. AD drugs have usually been used in acidic and hydrophilic solvents to supply moisture and prevent lipid defects. Ceramide is a typical treatment agent to regenerate the stratum corneum and relieve symptoms of AD. However, ceramide has limitation on direct use for skin because of its low dispersion properties in hydrophilic phase and side effects at excessive treatment. In this study, ceramide imbedded PLGA nanoparticles were developed with chitosan coating (Chi-PLGA/Cer) to overcome this problem. The chitosan coating enhanced initial adherence to the skin and prevented the initial burst of ceramide, but was degraded by the weakly acidic nature of skin, resulting in controlled release of ceramide with additional driving force of the squeezed PLGA nanoparticles. Additionally, the coating kinetics of chitosan were controlled by manipulating the reaction conditions and then mathematically modeled. The Chi-PLGA/Cer was not found to be cytotoxic and ceramide release was controlled by pH, temperature, and chitosan coating. Finally, Chi-PLGA/Cer was demonstrated to be effective at stratum corneum regeneration in a rat AD model. Overall, the results presented herein indicated that Chi-PLGA/Cer is a novel nanodrug for treatment of AD.

Ceramide is a key factor forming the stratum corneum layer that has a brickwork-like structure. Horny cells, which are dead keratinocytes, play a role in the formation of bricks, and lipid groups including ceramide adhere tightly to the lipophilic surface of horny cells. This layer is so dense and hydrophobic that substances rarely invade, which prevents inner moisture of the skin from being lost or evaporating^1^,^2^. Ceramide is known to effectively regenerate the layer, enhancing its aforementioned functions and relieving dehydration of skin and AD^3^,^4^,^5^. Ceramide also induces keratinocytic apoptosis with other oxidized phospholipids present in the stratum corneum that play a role in differentiating keratinocytes to horny cells and then regenerating the stratum corneum^6^. However, excessive treatment with ceramide not only causes irreversible cell death, but also leads to skin inflammation^7^,^8^. Excessive apoptosis of keratinocytes can lead to psoriasis and has the potential to cause infection in the loose stratum corneum^9^. For these reasons, temporal control of ceramide concentration is essential to proper skin regeneration. However, during treatment of AD with lipid groups, hydrophilic solvents are usually applied to supply moisture and prevent lipid defects^10^. In addition, the pH of skin afflicted with AD shows a slightly higher pH (around 6.0) than normal skin (pH 5.0 to 5.5), which invokes serine protease activity on the lesion, inhibiting its regeneration. This is usually treated by application of an acidic solvent or cream^11^. Nevertheless, the low solubility of ceramide in hydrophilic solvents makes it difficult to take temporal control over ceramide in AD^12^. Accordingly, ceramide nanodrugs for AD must have two major characteristics: 1) temporal control of release in acidic environments, 2) long-term storage and good-dispersion in hydrophilic solvent.

FDA-approved polylactic-co-glycolic acid (PLGA) has been applied to drug delivery, medical treatment, and tissue engineering due to its biodegradable and biocompatible characteristics^13^,^14^. Hydrophobic PLGA is adequate for imbedding hydrophobic macromolecules such as ceramide^15^. The coiled structure of PLGA at around 36.5 °C helps to efficiently and completely release encapsulated drugs^16^. Accordingly, colloidal carriers of PLGA have been widely applied as drug delivery systems. However, the hydrophobic nature of PLGA limits its application to skin treatment; accordingly, there have been efforts to enhance use of ceramide encapsulated with PLGA complex nanoparticles^17^. In addition, the coiled structure of PLGA which shrinks at around 36.5 °C, also leads to a drug burst in the early phase^18^,^19^. Therefore, it is still necessary to develop an efficient ceramide/PLGA nanodrug complex to effectively control temporal release and add positive functions for treatment of AD.

Biopolymers have also been studied as materials for nano-vehicles. Chitosan is an abundant natural polysaccharide obtained from deacetylization of chitin extracted from crustaceans and mollusks that is biocompatible and nontoxic, but antibiotic and antifungal^20^. Chitosan has gained attention owing to its potential for use as a drug carrier because of its positive charge, which enable it to easily approach to the cell membrane and imparts it with high formability and biodegradability^21^. Chitosan coating of nanodrugs is adequate to control drug release under acidic conditions since it dissolves well at around pH 5^22^. Chitosan coated PLGA nanoparticles have been investigated for use in methods of sustained drug release^23^. In addition, many studies have investigated interactions between chitosan and several lipids^24^. Hydrogen bonding regulates interactions between chitosan and lipids, leading to formation of a chitosan layer on the lipid layer by surface pressure^25^. However, few studies have investigated the interaction between chitosan and ceramide.

The present study was conducted to develop chitosan-coated ceramide/PLGA nanoparticles to overcome the major obstacles of ceramide treatment. Temporal control of ceramide/PLGA nanoparticles coated with chitosan was suggested to effectively control a release pattern which is determined by specific conditions of the target site such as pH and temperature. In this study, the *in-vivo* efficacy of the newly developed chitosan-coated nanodrug was evaluated in an *in-vivo* AD model system. Additionally, evaluations were conducted to verify that it was specifically controlled depending on pH and temperature conditions of the abnormal skin model. In parallel, thermodynamic analysis was conducted to investigate the formation mechanism of the chitosan-coated ceramide/PLGA nanoparticles, and safety of the nanodrug was demonstrated by an *in-vitro* cytotoxicity study using skin cells.

## Results

### Formation of Chi-PLGA/Cer

**Figure 1:**
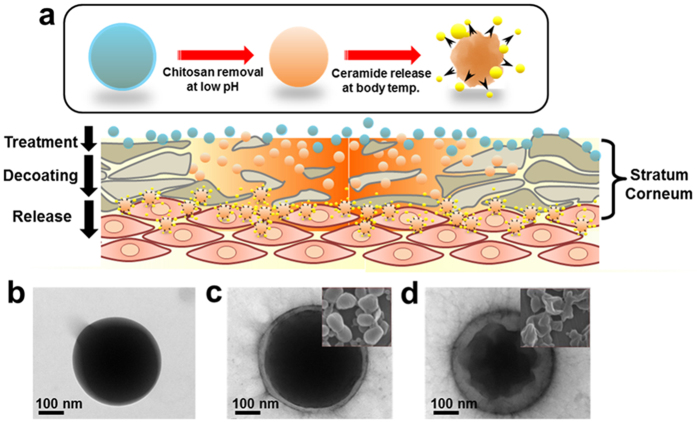
Schematic design of Chi-PLGA/Cer treatment on AD lesion (a) and confirmation of the shape of PLGA nanoparticles, chitosan coating, and shrinkage of PLGA by TEM and SEM (b, c, d). (**b**) A TEM image of non-coated PLGA nanoparticle. (**c**) A TEM image of coated PLGA nanoparticle and a SEM image of PLGA nanoparticles stored at 4 °C. (**d**) A TEM image of coated PLGA nanoparticle and a SEM image of non-coated PLGA nanoparticles stored at 36.5 °C.

Two obstacles to application of ceramide to AD were investigated: 1) temporal control of its release under acidic conditions, 2) long-term storage and good dispersion in hydrophilic solvent. To overcome the obstacles, the nanoparticles initially need to interact with around environmental condition and maintain a coating for a long period of time to prevent the inner molecule from leaking. To satisfy these requirements, nanoparticles with inner PLGA vehicles and outer chitosan coatings were formulated. In this system, the chitosan coating plays an especially important role in long-term storage. In Fig. 1, the PLGA nanoparticle was smooth sphere with ceramide. As shown in Fig. 1c,d, the surface of the Chi-PLGA/Cer was smooth and contained a packed coating layer at 4 °C and 36.5 °C (Fig. S1). TEM analysis confirmed that the chitosan coating was maintained, even if shrinkage of the inner PLGA nanoparticles occurred (Fig. 1d).

### Characterization of PLGA nanoparticles

**Figure 2:**
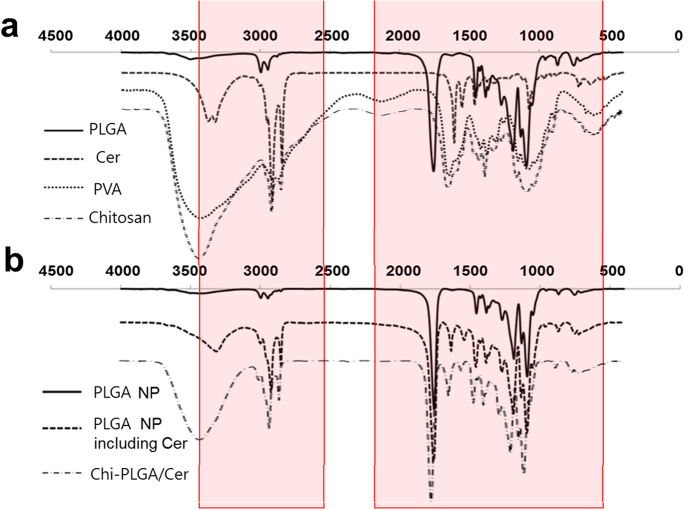
FT-IR spectrum for confirmation of the composition of nanoparticles. (**a**) Standard materials comprising the PLGA nanoparticles, (**b**) PLGA nanoparticles according to the presence or absence ceramide and chitosan coating.

Component analysis of PLGA nanoparticles and PLGA/Cer nanoparticles was performed by Fourier transform infrared spectroscopy (FT-IR). The FT-IR peaks of pure PLGA, ceramide, and PVA were comparatively analyzed as shown in Fig. 2. PLGA produced three main peaks. The absorption peak at 1757 cm^−1^ and 3000–2700 cm^−1^ indicates C=O (stretching) and C-H (stretching), respectively, while the absorption at 1500 cm^−1^ indicates the presence of C-H (deformation), C-H (wagging vibration) and C-O (ester) (stretching). The main peak of ceramide, which has an amide bond between sphingosine and fatty acid, was observed at 3350 cm^−1^ and 1560 cm^−1^. The absorption of PVA was detected at 3600–3300 cm^−1^ owing to a hydroxyl group and at 1300–1000 cm^−1^ due to a C-O (ether) group. FT-IR analysis of PLGA nanoparticles after lyophilization produced a graph with a shape identical to that of pure PLGA, while the graph of PLGA/Cer nanoparticles contained peaks of pure PLGA and pure ceramide without any loss of or addition of peaks. Additionally, FT-IR showed the major chitosan peaks in the result of Chi-PLGA/Cer (Fig. 2b).

The size, zeta potential, and dispersion of the nanoparticles were analyzed using a Z-analyzer (Table 1 and Fig. S2). The diameter of PLGA nanoparticles and PLGA/Cer nanoparticles was 207.8 ± 43.1 nm and 226.5 ± 30.5 nm, respectively. After coating the nanoparticles with 0.5 mg/ml of chitosan solution, the diameter of the PLGA nanoparticles and PLGA/Cer nanoparticles was 213.9 ± 30.8 nm and 211.4 ± 35.2 nm, respectively (Table 1). Zeta potential, which is a measure of the electric potential of the interfacial layer of a nanoparticle, determines the inter-particle repulsive force and stability in suspension. PLGA nanoparticles possessed a mean zeta potential of –46.86 ± 3.5 mV, which was caused by the ionized carboxyl group on the PLGA nanoparticles. PLGA/Cer nanoparticles had a mean zeta potential of –34.24 ± 6.7 mV due to ceramide addition. PLGA and PLGA/Cer nanoparticles had a mean zeta potential of +36.23 ± 2.7 and +49.31 ± 2.4 mV, respectively.

### Thermodynamic insights into adsorption of chitosan on PLGA/Cer nanoparticles

**Figure 3:**
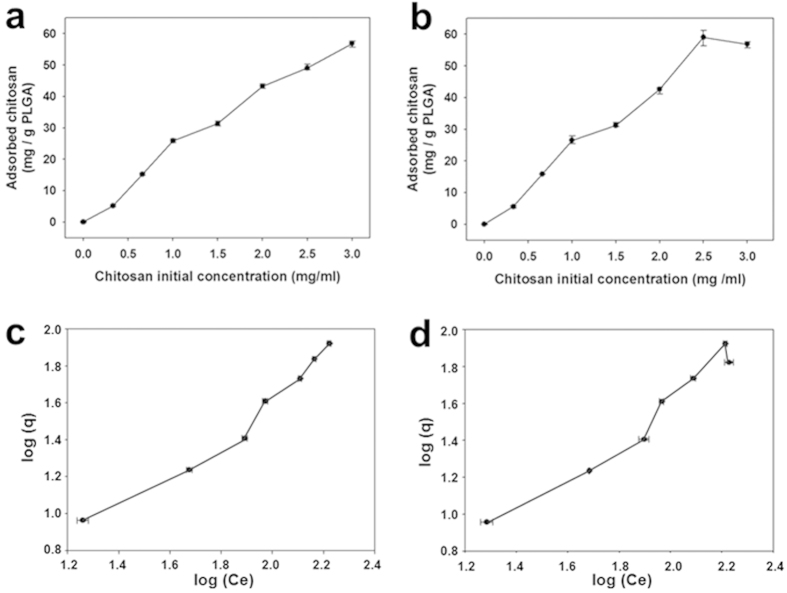
Chitosan adsorption quantification and modeling based on the quantification data. (a,b) Quantification of chitosan absorbed to PLGA nanoparticles according to the initial concentration of chitosan by fluorescamine protein assay. (**a**) No ceramide, (**b**) including ceramide. (**c**,**d**) Linear graph derived from the Freundlich isothermal model at 4 °C. (**c**) No ceramide, (**d**) including ceramide.

As the concentration of chitosan increased, the amount of chitosan coated on particle surfaces increased as well, while the thickness of the coating remained consistent after reaching a saturation point (Fig. 3). The amount of chitosan absorbed over the amount of PLGA increased from 0 mg to 80 mg chitosan/mg PLGA depending on the concentration of chitosan. To investigate the effects of ceramide on coating status, thermodynamic models (the Langmuir, BET, and Freundlich equations) were determined using the amount of absorbed chitosan (q) and the concentration (C_e_) at equilibrium at 4 °C. The experimental data were correlated with the Langmuir and BET models at low concentrations of absorbate, but these models could not explain the chitosan coating mechanism at high concentrations (Fig. S3). Conversely, the Freundlich model, which is based on an empirical adsorption isotherm equation, had an R^2^  = 0.95 (Fig. 3c,d). The model equation was generated using the amount of absorbed chitosan (q) per amount of PLGA and chitosan concentration (C_e_) in the equilibrium state. The n value indicates the affinity between chitosan and PLGA and nanoparticles, while the slope (1/n) from 0.1 to 0.5 represents strong adsorption. N values of 1.013 and 1.0027 were observed, indicating strong adsorption. In table 2, there were no significant differences in the q value, regardless of whether ceramide was added. Conclusively, the addition of ceramide does not affect a bonding between nanoparticle surfaces and chitosan.

### Controlled release of ceramide from nanoparticles depending on environments

**Figure 4:**
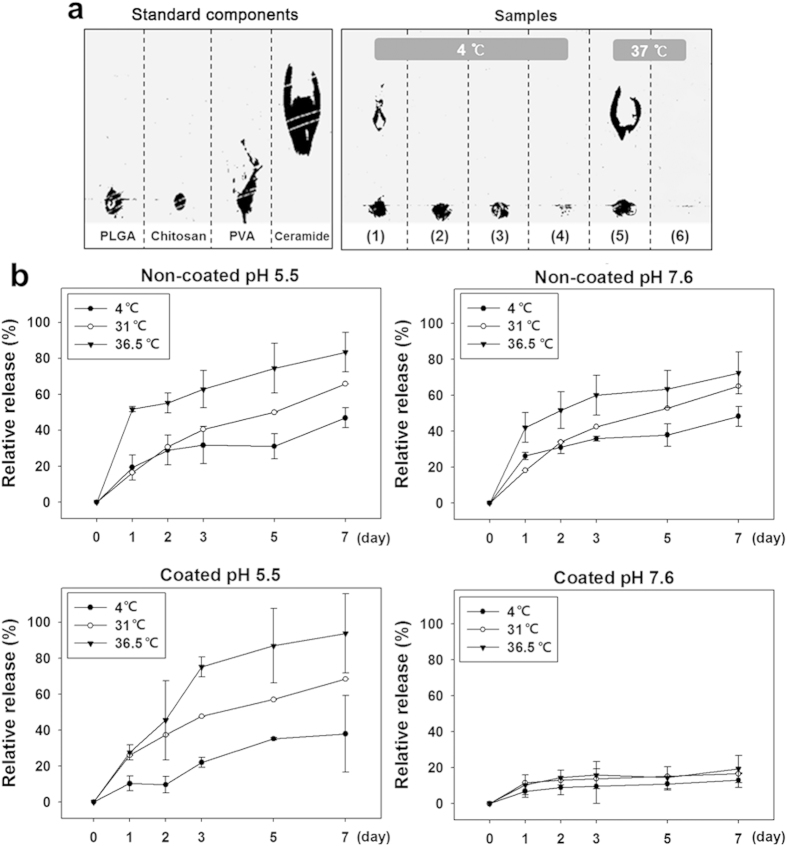
Qualitative and quantitative analysis of release of ceramide. (a) Qualitative analysis by TLC. The number on the figure refers to each sample: (1) non-coated PLGA nanoparticles (4 °C), (2) coated PLGA nanoparticles (4 °C), (3) supernatant of non-coated PLGA nanoparticles (4 °C), (4) supernatant of coated PLGA nanoparticles (4 °C), (5) supernatant of non-coated PLGA nanoparticles (36.5 °C) and (6) supernatant of coated PLGA nanoparticles (36.5 °C). (**b**) Quantitative analysis and conformation of release pattern by HPLC for 7 days. Values are expressed as the mean ± S.D., P-value was defined as *<0.05, **<0.01, ***<0.001 (n = 3).

As shown in Fig. 4a, the release of ceramide from PLGA nanoparticles was confirmed by TLC. Nanoparticles are composed of PLGA, chitosan, and ceramide. TLC was performed using a mixture of chloroform and methanol as the mobile phase. In the case of PLGA nanoparticles containing ceramide, released ceramide was moved and became black. However, in the case of the chitosan coated PLGA nanoparticles, movement of ceramide was not observed. In the above results, ceramide was located within the chitosan membrane, and the release of ceramide did not occur before the coating was removed. In the case of samples stored at 4 °C, ceramide was not released, regardless of the chitosan coating. For samples stored at 31 °C and 36.5 °C, release of ceramide only occurred for non-coated PLGA nanoparticles. Even if shrinkage of PLGA nanoparticles occurred at 36.5 °C, release of ceramide did not occur unless the chitosan coating was removed. These results are confirmed by the data presented in Fig. 4a.

HPLC revealed how much ceramide was included in the nanoparticles sample and released from the samples. In the case of the control, the nanoparticle showed a 72.51 ± 0.64% load ratio of ceramide. In the fabrication step, 20 mg of ceramide was added to 50 mg PLGA and 14.5 ± 0.32 mg ceramide remained in the PLGA nanoparticles. In the case of non-coated PLGA nanoparticles, more ceramide was released at high temperature, regardless of pH. In the case of chitosan coated PLGA nanoparticles, if the chitosan coating was not removed, the amount released remained under 13.3 ± 0.07%, regardless of temperature. While most ceramide was released in 1 day when the chitosan coating was removed, ceramide was released slowly over three days when the chitosan coating was not removed (Fig. 4b). Ceramide was not released over the saturation level under storage conditions (4 °C) over a week period.

### Nanoparticle penetration assay using *ex-vivo* skin tissue

To observe the nanoparticle penetration into skin tissue, FITC-labeled Chi-PLGA nanoparticles were treated onto the rat skin tissue. As shown in Fig. S4, the nanoparticles were penetrated into stratum corneum and cumulated at the boundary between epidermis and dermis. However, the nanoparticles were not shown at dermis layer.

### Confirmation of non-toxicity of the nanoparticle by MTT assay

**Figure 5:**
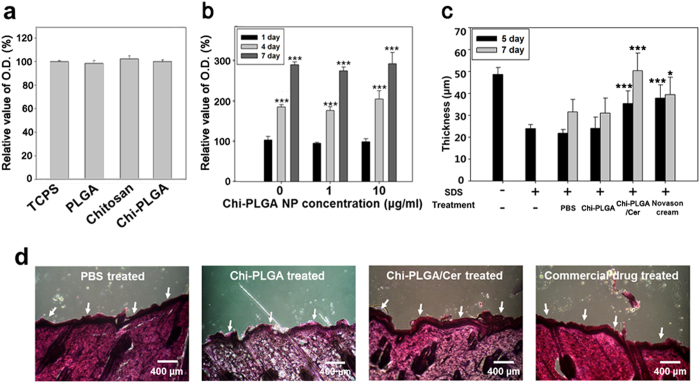
*In-vitro* assay and *in-vivo* assay to evaluate Chi-PLGA/Cera efficacy. (**a**) Confirmation of cell cytotoxicity. (**b**) Evaluation of cell viability of dermal fibroblasts. The values are expressed as the mean ± S.D. There was no significant difference between samples (n = 3). (**c**) The quantified thickness of stratum corneum using cross-section images of (**d**). Each column was compared with PBS treated sample at the same day. P-value was defined as *<0.05, **<0.01, ***<0.001 (n > 7). (**d**) Cross section images of PBS, Chi-PLGA, Chi-PLGA/Cer and Novason cream treated samples at day 7.

Evaluation of the cytotoxicity of the material constituting the nanoparticles confirmed that they do not affect cell viability (Fig. 5a). Chitosan and PLGA are already used as biocompatible materials, and their composite has no cytotoxicity. Moreover, chloroform was removed and no reaction between chitosan and PLGA occurred during the formation of PLGA nanoparticles. Evaluation of the cellular activity for 7 days revealed no significant differences between cells cultured in normal media and culture media containing PLGA nanoparticles (Fig. 5b).

### Evaluation of *in-vivo* efficacy of ceramide

We selected 4 week old SD-rats because they are useful for simulating AD in infants, children and adolescents. Stratum corneum is known to be dried by SDS, which results in the layer becoming dehydrated and the composition of lipids in the layer changing. After treating SDS, the stratum corneum was removed and observed by microscopy (Fig. S5). On day 5, the stratum corneum in PBS treated samples had not become dense and was apart from the surface, while samples treated with Chi-PLGA/Cer and the commercial product showed some recovery of the stratum corneum (Fig. S5). On day 7, tissue similar to typical tissue arose in samples that had been treated with Chi-PLGA/Cer (Fig. 5d). Moreover, this recovery was faster than self-reproduction and nearly the same speed as that observed for the commercial product.

The trends shown in the images were reaffirmed by the quantification graph (Fig. 5c). Before SDS treatment, the thickness of the stratum corneum was 48.97 ± 3.54 μm; however, the thickness decreased to 23.90 ± 1.90 μm after treatment. At day 5, the thickness of the PBS treated stratum corneum was 21.84 ± 1.70 μm, the Chi-PLGA treated layer was 24.00 ± 5.20 μm, the Chi-PLGA/Cer treated layer was 35.41 ± 5.72 μm and the commercial product treated layer was 37.87 ± 6.08 μm. Based on these findings, the PBS treatment did not help to regenerate the layer, while dramatic regeneration with no significant differences was observed in response to the other treatments. At day 7, the layer was restored to 31.51 ± 5.70 μm (PBS), 31.23 ± 6.70 μm (Chi-PLGA), 50.40 ± 8.00 μm (Chi-PLGA/Cer) and 39.47 ± 7.84 μm (commercial product). These findings indicate that PBS led to slight regeneration, while the Chi-PLGA/Cer treatment induced the best results, restoring the samples to almost normal levels. The regeneration of stratum corneum was confirmed by filaggrin staining. The filaggrin was dispersed well in the stratum corneum when the Chi-PLGA/Cer and Novason cream were treated. Otherwise, it was hard for filiagrin to be observed in the stratum corneum without therapeutic molecules such as ceramide (Fig. S6). To evaluate if nanoparticle treatment induces inflammation, inflammation markers (MCP-1 and TNF-α) were analyzed by using qRT-PCR. At day 7, MCP-1 and TNF-α showed the similar expression level whether the ceramide was treated or not. Other markers were not elevated significantly (Fig. S7).

## Discussion

To relieve AD, acidic and hydrophilic solvents are required to provide moisture and prevent lipid defects. Although ceramide needs to be utilized through solvent for treatment, the ceramide has limitations on release into stratum corneum because of its lipophilic properties. In the present study, a nanodrug has been developed and demonstrated that it is capable of carrying and dispersing ceramide at neutral pH whereas controllably release the embedded ceramide under the acidic conditions of lesions of AD or psoriasis.

The nanoparticle is evaluated that it is able to carry ceramide and disperse it well into hydrophilic phase for application. To overcome this problem, PLGA should be mixed with ceramide well and be formulated to nanoparticles. The results of FTIR (Fig. 2) showed that ceramide existed as a form of nanoparticles in PLGA, confirming the carrier potential of the developed system. PVA, which was used as a stabilizer^26^, was almost not shown in the PLGA nanoparticles. After measurement about nanoparticles, we performed the FTIR to the chitosan coating. Based on the analysis, any peak from additional chemical reaction was not observed at the sample with the chitosan coating (Fig. 1c), and we asserted the coating would be formed by electrostatic interaction between chitosan and PLGA/ceramide. In addition, the result showed that no extra chemical reactions occurred. These findings demonstrate that PLGA nanoparticles only include the initial ingredients, without any byproducts of chemical reactions. For the result, the nanoparticles were only composed of bio-usable molecules.

Following chemical evaluation, the nanoparticle dispersion was evaluated by a zeta potential. In table 1, dispersions of the nanoparticles were demonstrated by the value of zeta potential. The value represented electric static force of particle surface and this force made push the each particle out. The pushed nanoparticle was hard to aggregate or precipitate and finally located equally in solution. The dispersion was defined with the standard deviation of diameter. The small standard deviation showed that the nanoparticle and coating were uniformly formulated and also showed that nanoparticle was present without aggregation. It showed the dispersion of nanoparticles as an evidence of no aggregation. As shown in Fig. S2, distribution of the particle size was shown as sharp one-pole pattern at each sample. If aggregation was dominantly occurred, it would have shown wide bell shape with high average value or more than double poles. Thus, the sharp distribution pattern and one-pole shape supported the fact that there were no dominant aggregation, and the particles were dispersed well despite of addition of the ceramide.

After defining the distribution, we characterized the chitosan coating, an important part of the system. The chitosan coating prevents the imbedded drug from leaking before appropriate conditions are met. The average diameter was not increased significantly after chitosan coating, which may have occurred owing to the strong electrostatic attraction, such as hydrogen bonds, between PLGA and chitosan. During the coating, chitosan is present in the sub-layer, which is the layer above the basal layer (PLGA or PLGA/Cer), and chitosan molecules are in contact with the basal layer. Contacted molecules generate surface pressure by hydrogen bonds and form packed coating layers^27^. This surface pressure by coating would decrease the diameter of coated nanoparticles. This was demonstrated by the occurrence of great differences in zeta potential between non-coated and coated nanoparticles, which showed that sufficient electrostatic attraction existed on the nanoparticle surfaces to pack the chitosan coating layer. The strong negative charges of both PLGA and PLGA/Cer nanoparticles in neutral solution impart them with inter-particular repulsive force and better dispersion stability. However, after chitosan coating, the mean zeta potential of nanoparticles increased dramatically. Since chitosan is a cationic polymer containing amino groups, it forms hydrogen bonds with surfaces of the negative nanoparticle, causing chitosan-coated PLGA nanoparticles to have a positive zeta potential. Additionally, these particles appeared to have a limit to the thickness of the coating at higher chitosan concentrations^29^. Conclusively, chitosan coating on nanoparticles has sufficient rigidity and density during nanodrug storage.

Analysis of these zeta potential changes revealed that the Freundlich model was imitated well in our system. Chitosan adsorption has a linear function with concentration of chitosan and was correlated at a wide range in the Freundlich model. Conversely, the Langmuir and BET models were only imitated at low concentrations of chitosan (0–0.33 mg/ml; Fig. S3). Since the Langmuir model assumes adsorption of a single layer and the BET model is primarily derived from the Langmuir model, the BET model also has similar limitations. However, the Freundlich model is based on an empirical equation and imitates multi-layer adsorption for both a homogeneous and heterogeneous surface well. Accordingly, the Freundlich model did not show the error at low concentrations, indicating that multiple layers of chitosan were observed on the PLGA surface. In the model, the n value (adsorption intensity) was over 1, indicating that the chitosan tends to induce the reaction to proceed toward the adsorption of PLGA under isothermal conditions^28^,^29^. Overall, parameters related to chitosan absorption remained similar, regardless of the inclusion of ceramide. Based on these findings, we confirmed the mechanism of chitosan coating. According to the mechanism, coating thickness was not changed when we imbedded ceramide in PLGA nanoparticles. The chitosan coating of PLGA nanoparticles is stable and its thermodynamic stability leads to benefits during its storage. Use of this mathematical approach facilitated our evaluation of the design and robustness of the developed system. In conclusion, chitosan adsorption is correlated with its concentration, and this correlation is well-defined through isotherms for applying to form proper coating on the PLGA nanoparticles. This stable chitosan coating shows potential for use as a layer for long-term storage.

After characterization of the nanoparticle, we evaluated the potential of the nanoparticle about the temporal control for efficient release at target site. To simulate removal of this stable coating in specific lesions, pH and temperature were changed. The results of TLC and HPLC demonstrated properties of interaction with acidic solvents and ceramide release control. Even though the coating layer had thermodynamic stability, low pH removed the chitosan layer by direct dissolution of the molecules into solvent. Additionally, we showed that chitosan and the surface interacted via electrostatic attraction, but that at low pH, the positive-rich condition weakened the interaction. For this reason, chitosan could be removed from the nanoparticle surfaces at low pH as the same pH as peri-lesion of AD^30^. After removing chitosan, the imbedded ceramide was ready to be released on skin. On the other hand, ceramide in Chi-PLGA/Cer nanoparticles was prevented from being released at neutral pH by the chitosan coating and protected from oxidation and phase separation with hydrophilic solvent. However, even if the pH conditions were met, adequate temperature was also required for the full release of ceramide. PLGA vehicles decreased release of ceramide significantly at 4 °C, but ceramide was released at 31 °C and 36.5 °C. As a result of ceramide release assay, especially, it was proposed that the shrinkage of nanoparticles and release of ceramide were closely related, where release of the ceramide was enhanced at 36.5 °C and shrinkage took place at the same temperature of 36.5 °C (Figs 1 and 4). Therefore the release pattern was shown to increase at specific condition of the temperature at which shrinkage also affects the release pattern. Even though it was indirect confirmation, these results implied to their relationship. Based on the observations, we suggest that shrinkage plays a distinct role of temporal control for release of the payloads depending on characteristics of the nanoparticles and micro environment such as pH, temperature and solvents^19^,^20^. We also measured the release pattern at 31 °C as the same temperature of rat *in-vivo* AD model. The release pattern was similar to that at 36.5 °C, which verified that the nanoparticle release test in the rat AD model system is applicable to AD lesion of human body. PLGA/Cer nanoparticles were also subjected to long-term storage to confirm that ceramide was not released under storage conditions (4 °C). The storage properties implied the rigidity of the chitosan coating and that this rigidity was derived from hydrogen bonds. Under insoluble conditions, only electrical neutralization prevents chitosan decomposition from the basal layer. In order to dissolve molecules to overcome electrical neutralization, pH changes or voltage supplements are required^31^. However, under storage conditions, there were no additional condition changes and chitosan coating maintained its strong bond between basal layers to prevent the release of inner molecules. For this reason, the multi-encapsulation of PLGA and chitosan appeared to have a synergistic response to the pH and temperature. These properties can be used to enable application of a nanodrug to a skin lesion under the optimal conditions, and this responsive nanodrug system is suitable for treatment of AD.

Through the release pattern we defined the nanocomposite as a carrier able to release payloads steadily and long-termly. However, it was not enough to say that we overcome the other obstacle to use ceramide in AD; efficient transfer of ceramide into stratum corneum. For the efficient transfer, the ceramide should be released as much from the carrier. As shown in Fig. 4b, initial burst was observed due to the character of PLGA. However, ceramide in coated carrier released more slowly than ceramide in non-coated carrier. The nanodrug has a potential to steady and long-term release, but the nanoparticles are easily removed from outer surfaces of stratum corneum by daily activities and washing. For this reason, the nanoparticles is better to be located the site to preserve it from the activities and to secure time to fully release such as inner side of stratum corneum. In case of ceramide, there is one more requirement that the carriers should not penetrate over epidermis. The ceramide occur apoptosis and it would help to dense stratum corneum but it is a hazard to other site. The nanoparticle penetration is shown in Fig. S4. As shown in the figure, nanoparticle was cumulated at the epidermis without penetration into dermis of the *ex-vivo* skin tissue. We could assert two possibilities to the penetration; 1) the loose stratum corneum of AD compared with dense layer of normal skin would be the cause of the higher penetration rate, or 2) penetration through aqueous route in intercellular space of skin barrier by chitosan coating enhancing hydrophilicity^32^. Conclusively, the nanoparticle accumulation decreases loss of nanodrug before penetrating into stratum corneum and then the most ceramide in the nanoparticles was able to be transferred to target site after penetration.

Safety of nanodrug was evaluated before *in-vivo* efficacy assays. As shown in Fig. 5a,b, there were no significant differences among the treated samples. Additionally, there were shown the same patterns and levels of cell viability for 7 days. These results implied that the nanoparticles did not result in release of any toxic molecules. Indeed, safety of each component of the nanoparticles was already evaluated for human. The nanodrug already checked that it was a mixture of the bio-usable materials without other reaction by FT-IR (Fig. 2).

Finally, the efficiency of this multi-responsive nanodrug was tested *in-vivo* after confirming its non-excessive release in an *in-vitro* assay. As shown in Fig. 5d, the regenerated stratum corneum differed among samples after SDS treatment. Specifically, treatment with ceramide nanoparticles and commercial product led to dense stratum corneum and restored its thickness relative to the normal thickness. In contrast, PBS and Chi-PLGA without ceramide treated sample had a loose layer or no layer, and the stratum corneum was not regenerated until day 5 after samples treatment. This loose structure resulted in easy removal of horny cells during the experiments. Ceramide works as cement in the stratum corneum; therefore, horny cells would not be able to construct the separation layer by themselves owing to the absence of ceramide^3^. On the other hand, the epidermis treated with ceramide nanoparticles was restored to the level before SDS treatment. Ceramide differentiates keratinocytes to horny cells and supplies the material used to generate the stratum corneum^4^,^5^ A nanodrug was effective as the betamethasone valerate, which is also known to lead to keratinocyte apoptosis as a steroid^33^. Overall, the ceramide treated epidermis was regenerated more quickly than during natural healing. In addition, this regeneration level was similar to that obtained by the commercial product, even though the ceramide-nanoparticles have not yet been optimized. As shown in Fig. S6, the filaggrin, a filament associated protein, was observed and it was well-dispersed in regenerated stratum corneum. Filaggrin plays a role in maintaining homeostasis of epidermis and constructing lipid packed layer of stratum corneum by its lipid enveloping function^34^. The filaggrin dispersion implied that the nandrug recovered not only the thickness of stratum corneum but also dense structure of stratum corneum. In addition, the nanodrug was evaluated its safety by expression levels of inflammatory factors (MCP-1 and TNF-α). For the Fig. S7, any marker was not over-expressed when the nanodrug was treated compared to other sample. For the result, we asserted that the nanodrug did not induce inflammation^35^. Taken together, these findings suggest that our nanoparticles have the potential for use as a carrier of ceramide and can be applied to treat AD. Conclusively, these biological results showed that ceramide imbedded in nanoparticles was successfully released from inner PLGA nanoparticles. To release ceramide, the nanodrug must respond to the conditions typical of skin; namely, a low pH for removal of the chitosan coating and body temperature for increasing ceramide release. In the present study, the nanodrug system worked well *in-vivo*, demonstrating its potential for use as an effective nanodrug for AD.

In this study, we suggested a potential nanosized drug delivery system for ceramide to relieve AD and psoriasis. This carrier operates through the controlled release of ceramide based on skin pH and temperature. Chi-PLGA/Cer nanoparticles were formulated through oil-in-water emulsion and subsequent chitosan coating on the surface of the PLGA particles. The particles generated using this method had homogeneous size and shape. The chitosan coating mechanism was confirmed through the adsorption isotherm equation. Chitosan coated PLGA nanoparticles were reproducibly formed and the nanoparticles maintained their shape and chemical structure under storage conditions. However, ceramide was released by dissolved chitosan, and the PLGA shrank at pH 5.5 and 36.5 °C, which are typical of actual skin. Therefore, the initial burst of PLGA nanoparticles was compensated for by the chitosan coating. Overall, we created a drug carrier system capable of controlled release of ceramide that showed no toxicity to dermal fibroblasts and effectively regenerated the stratum corneum in the psoriasis animal model. These findings suggest the possibility for development of release-controllable drug carriers for use in the cosmetics industry and in dermatological preparations.

## Online Methods

### Formulation of chitosan coated PLGA nanoparticles

Poly (lactic-co-glycolic) acid (PLGA: Sigma-Aldrich, St.Louis, MO, USA) nanoparticles were fabricated through oil-in-water emulsion methods followed by evaporation of the solvent. Specifically, 50 mg of PLGA were dissolved in 1 ml of chloroform and the solution was immersed into 10 ml of 0.5% w/v poly(vinyl alcohol) (PVA: Sigma-Aldrich, St.Louis, MO, USA) in distilled water. Next, 20 mg of ceramide were added to the PLGA solution to generate ceramide imbedded PLGA nanoparticles. PVA, which is a nonionic and hydrophilic polymer, was used as stabilizer for dispersion of PLGA. The emulsified solution was homogenized for 15 sec at 1500 W using an ultrasonicator (STH-2000S, SL Science, South Korea), during which time the solution changed from transparent to a milky translucent color. Next, the chloroform was evaporated from the solution from the prior step by magnetic stirring at 100 rpm for one day. All processes were performed at 4 °C. To obtain fully formed nanoparticles, the sample was subjected to ultracentrifugation (Hanil, South Korea) at 20,000 × g for 20 min at 4 °C, then dissolved in 10 ml of 0.5% w/v PVA solution in distilled water. The aggregated particles were removed using a 0.45 μm membrane filter and the final solution was stored at 4 °C until assayed. To coat chitosan onto the PLGA nanoparticles, chitosan solution in 1% acetic acid was prepared at various concentrations (0, 0.11, 0.22, 0.33, 0.5, 0.66, 0.83, 1 mg/ml). Chitosan solution was then added to the PLGA nanoparticle solution with magnetic stirring for 2 h at 4 °C, after which the chitosan coated nanoparticles were collected and stored by the same method as described above.

### Morphological analysis of PLGA nanoparticles by SEM and TEM

The shape and chitosan coating of fabricated PLGA nanoparticles were confirmed by SEM and TEM. To accomplish this, fabricated nanoparticles were completely dried in a vacuum and then coated with Pt before observing via SEM. In case of TEM, the nanoparticles were dropped on the TEM grid and then stained with 1% uranyl acetate solution in the dark. Next, the sample was washed with distilled water and the drop on the grid was removed. The grid was then kept in a vacuum for one day until it was completely dry, after which nanoparticles were observed using a TEM.

### Detection of ceramide in nanoparticles

Ceramide in PLGA nanoparticles was characterized by Fourier transform infrared spectroscopy (VERTEX 80V, Bruker, Billerica, MA, USA). The nanoparticles were compared with each standard molecule (PLGA, PVA, ceramide), and samples were prepared by mixing KBr and grinding.

### Size and zeta potential

The size, dispersion and zeta potential of the nanoparticle solution were analyzed by dynamic light scattering using a laser particle size analyzer (Mastersizer 2000, Malvern, UK). Sample solution was prepared by 10-fold dilution of nanoparticle solution with distilled water.

### Adsorption of chitosan and adsorption isotherm equation

Quantitative analysis of chitosan on the surface of the PLGA nanoparticles was conducted by fluorescamine protein assay. Fluorescamine can react with the amine functional group of chemicals, resulting in fluorescent products. Chitosan was the only chemical with an amine group among the nanoparticles; therefore, the amount of coated chitosan was quantified by a fluorescamine assay. Briefly, 20 μl of the sample solution was mixed to 4 μl of 2 mg/ml fluorescamine solution in dimethyl sulfoxide (DMSO, Sigma-Aldrich, St.Louis, MO, USA) to react for 3 h in the dark. After reaction, the fluorescence intensity was detected at 390 nm excitation and 515 nm emission wavelengths using a Varioskan flash spectral scanning reader (Thermo Fisher Scientific, Waltham, MA, USA). The amount of chitosan attached to the nanoparticles was calculated by the difference between the chitosan remaining after the coating reaction and the initial amount of chitosan. The fluorescence intensity was converted to the concentration of chitosan by comparison with a standard curve of pure chitosan. The amount of chitosan attached to the particle was then applied to the adsorption isotherm equation (Equation 1):


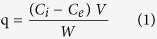


where, q is the amount of chitosan attached to the PLGA nanoparticles per mass of the nanoparticle, V is volume of nanoparticle solution, W is the mass of the nanoparticles, and C_i_ and C_e_ are the concentration of chitosan during the initial state and equilibrium state, respectively.

The Langmuir (Equation 2)^36^, BET (Equation 3)^37^, and Freundlich (Equation 4)^29^ models were derived from the isotherm equation to apply the experimental model and verify the adsorption mechanism.


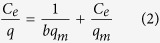



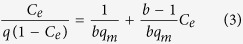



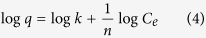


In these equations, q, q_m_ and K are the adsorption capacity, and b and n are the adsorption strength. The results obtained by each isotherm equation were expressed with linear equations.

### Assay of ceramide release by thin layer chromatography

Thin layer chromatography was used for qualitative confirmation of the release of ceramide from PLGA nanoparticles. The mixture of chloroform and methanol at a ratio of 6:4 was used for the mobile phase. Briefly, the sample was dropped on a TLC plate using a needle and spread, then allowed to stand for 30 min. Next, 10% sulfuric acid was applied to the TLC plate, after which it was heated at 150 °C for 1 hr, at which time separation bands appeared. Transport of the composition of the nanoparticles including PLGA, chitosan, PVA and ceramide was confirmed under the conditions described above, after which the release of ceramide from PLGA nanoparticles was confirmed.

### Quantitative analysis of ceramide and confirmation of release pattern

Quantitative analysis was performed using high performance liquid chromatography to confirm the release of ceramide from fabricated PLGA nanoparticles. A C18 polymer column (*W*: 4.6 mm, *L*: 15 cm) was used with a mixture of chloroform and methanol at a ratio of 19:1 applied for the mobile phase. Flow rate and pressure were maintained at 0.6 ml/min and 780 psi, respectively, and 10 μl of sample was injected at 30 °C. The initial encapsulation efficiency was calculated from the supernatant after nanoparticle formulation. Released ceramide was quantified and the release pattern of chitosan coated or non-coated PLGA nanoparticles stored at 4 °C, 31 °C and 36.5 °C (pH 5.5 and 7.4, respectively at each temperature condition) were analyzed on day 1, 2, 3, 5 and 7. Chromatography was performed after 10 ml of sample was concentrated 10-fold by ultracentrifugation. The supernatant was subsequently dried under vacuum, dissolved in 10 μl of mobile phase and injected into an injection port of the HPLC.

### *In-vitro* assay using primary dermal fibroblasts from rat

Dermal fibroblasts were isolated from skin tissue extracted from 1-day-old SD rats (DBL, South Korea). Extracted skin was then washed with PBS, after which blood vessels in the dermis were removed using tweezers and the sample was again washed with PBS. The dermis and epidermis were subsequently separated by reaction with dispase (1 mg/ml) (Gibco, Grand Island, NY, USA) in PBS solution at 4 °C for one day. The separated dermis was again reacted with collagenase 2 (1 mg/ml) (Gibco, Grand Island, NY, USA) in PBS solution at 4 °C for one day, after which cell debris was removed by passing the sample through a 70 μm strainer (BD Falcon, Franklin Lakes, NJ, USA). Next, dermal fibroblasts were cultured in DMEM-high glucose culture media (Gibco, Grand Island, NY, USA) containing 10% FBS (Gibco, Grand Island, NY, USA) and 1% antibiotic-antimycotic (Gibco, Grand Island, NY, USA) in a 5% CO_2_ incubator. Passage 3 dermal fibroblasts were prepared by subculture for all experiments. To measure the cytotoxicity of the nanoparticles, chitosan, PLGA nanoparticles and chitosan coated PLGA nanoparticles were incubated in culture medium for 24 hr. The fibroblasts were cultured in a 96-well plate with a concentration of 3 × 10^3^ cells/well for 1 day, after which the pre-treated culture media was loaded. To measure the cell viability, cells were seeded in a 24-well plate with a concentration of 1 × 10^4^ cells/well and cultured with media containing Chi-PLGA/Cer at a concentration of 0, 1 and 10 μg/ml for 7 day. MTT assay was used to evaluate the cell cytotoxicity and cell viability of the samples. Briefly, DMEM-H media containing MTT (Sigma-Aldrich, St.Louis, MO, USA) at a concentration of 0.5 mg/ml was prepared. Media in the 24-well culture plate was then removed and washed with PBS three times, after which 500 μl of MTT solution was added and incubated in a CO_2_ incubator for 1 hr. The MTT solution was then removed and DMSO 1 ml was added. The produced formazan was subsequently dissolved by stirring for 1 hr at room temperature. Finally, DMSO solution containing dissolved formazan was transferred to a 96-well plate and the optical density was measured using a micro plate reader (Tecan, Australia) at 540 nm.

### Ethical statement

All animal studies were carried out in accordance with the Guide for the Care and Use of Laboratory Animals recommended by the National Institutes of Health and ARRIVE guidelines (http://www.nc3rs.org/ARRIVE). All animal experiments were approved and conducted in accordance with the INHA-IACUC (Inha University-Institutional Animal Care and Use Committee) guidelines (Approval Number INHA 150924-381). In addition, all the procedures for animal experiments were performed in compliance with the approved IACUC-INHA protocol.

### Nanoparticle penetration assay using *ex-vivo* skin tissue

For *ex-vivo* assay, tissue was secured from back of SD-rat aged 4 weeks after shaving. 1 g of Fluorescein isothiocyanate (FITC, Sigma-Aldrich, St.Louis, MO, USA) was dissolved in 1 ml of 200 mM sodium bicarbonate solution (Thermo Fisher Scientific, Waltham, MA, USA). 1 ml of the Chi-PLGA solution from above steps was used. The Chi-PLGA was collected by centrifuge and it was mixed with 1 ml of FITC solution for 1 hr. After the reaction, Chi-PLGA was washed with distilled water and finally dispersed in PBS. Filter paper was cut into 1 cm^2^ and put on the secured tissue. The filter paper soaked 100 μl of FITC labelled Chi-PLGA solution and incubated for 2 hr at 36.5 °C. After PBS washing, the tissue was sectioned with cryostat. The tissue sections were observed at 465 nm of excitation and 545 nm of emission wavelength.

### *In-vivo* assay of the efficacy of released ceramide

An *in-vivo* assay was performed using SD-rats aged 4 weeks to evaluate the efficacy of released ceramide. The hair was removed from the rat’s backs, after which 10% sodium dodecyl sulfate (Sigma-Aldrich, St.Louis, MO, USA) in distilled water was applied for 2 days to generate a psoriasis model. On day 3, the rat’s backs were divided into four parts and treated with PBS, 5% ceramide solution in PBS, 20% Chi-PLGA in PBS, 20% Chi-PLGA/Cer in PBS, and a commercial product (Novason Cream; Green Cross, South Korea). Next, the back skin of the rats was extracted on day 5 and 7 after treatment and recovery of psoriatic skin was confirmed by H&E staining. Briefly, extracted skin tissue was fixed in 4% paraformaldehyde solution overnight and then treated with 30% and 50% sucrose serially until the skin tissue was settled. An OCT block of each skin tissue sample was then made using OCT compound (Sakura, Netherlands). The OCT blocks were subsequently sliced to 20–25 μm using a cryostat (HM 505 N, Microm, UK) at −40 °C, after which the skin sections were stained by H&E. Remaining OCT compound was removed by washing with PBS, then stained with hematoxylin (Sigma-Aldrich, St.Louis, MO, USA) for 8 min. After washing with PBS, the sections were stained with eosin-phloxine solution (Sigma-Aldrich, St.Louis, MO, USA) for 1 min and then washed with PBS. Stained sections were subsequently observed by optical microscopy, after which the recovery of the stratum corneum was compared. The thickness of each sample at day 5 and 7 was quantified from the microscopic images using image J. In addition, we checked the regeneration efficacy of stratum corneum through filaggrin, the component protein of stratum corneum to maintain its density. The sections were stained with anti-filaggrin antibody (Abcam, UK) and observed with confocal microscopy. In case of the inflammation assay for evaluating the nanodrug safety, the tissue was used as the same tissue as the efficacy assay. The mRNA of each tissue sample was extracted from the secured tissue using trizol and RNeasy Plus mini kit (Qiagen, Netherland). From the mRNA, the inflammatory marker (MCP-1 and TNF-α) were analyzed using qRT-PCR (Qiagen, Netherland). Each expression level of the markers was compared with β-actin expression level.

### Statistical analysis

All experiments were performed in triplicate and analyzed by t-assay using the Sigma-Plot program. The results are expressed as the mean ± S.D. (standard derivation).

## Additional Information

**How to cite this article**: Jung, S.-M. *et al.* Thermodynamic Insights and Conceptual Design of Skin-Sensitive Chitosan Coated Ceramide/PLGA Nanodrug for Regeneration of Stratum Corneum on Atopic Dermatitis. *Sci. Rep.* **5**, 18089; doi: 10.1038/srep18089 (2015).

## Supplementary Material

Supplementary Information

## Figures and Tables

**Table 1:** Size and zeta potential of nanoparticles according to the chitosan coating and inclusion of ceramide.

Sample	Diameter	Z-potential
Empty PLGA nanoparticles	207.8 ± 43.1 nm	−46.86 ± 3.5 mV
PLGA nanoparticles containing ceramide	226.5 ± 30.5 nm	−34.24 ± 6.7 mV
Empty Chi-PLGA NP	213.9 ± 30.8 nm	36.23 ± 2.7 mV
Chi-PLGA nanoparticles containing ceramide	211.4 ± 35.2 nm	49.31 ± 2.4 mV

**Table 2:** The parameter values derived from adsorption isotherms.

Isotherm equation	Chi-PLGA NP		Chi-PLGA NP with ceramide	
	Adsorption intensity	Regression coefficient	Adsorption intensity	Regression coefficient
Langmuir	q_m_ = 56.1798	0.9367	q_m_ = 60.9756	0.9414
BET	q_m_ = 54.3478	0.9388	q_m_ = 59.5238	0.9435
Freundlich	k = 0.6256	0.9584	k = 0.3936	0.9583
