# Supplementary material for: Thermodynamic Insights and Conceptual Design of Skin-Sensitive Chitosan Coated Ceramide/PLGA Nanodrug for Regeneration of Stratum Corneum on Atopic Dermatitis
Source: Sci Rep. 2015 Dec 15;5:18089. doi: 10.1038/srep18089 (PMC4678456; doi:10.1038/srep18089)
Supplement: Supplementary Information [file srep18089-s1.pdf]

Thermodynamic Insights and Conceptual Design of Skin-Sensitive Chitosan Coated Ceramide/PLGA Nanodrug for Regeneration of Stratum Corneum on Atopic Dermatitis

Supplementary Information

Sang-Myung Jung[+], Gwang Heum Yoon[+], Hoo Chul Lee, Moon Hee Jung, Sun Il Yu, Seung Ju Yeon, Seul Ki Min, Yeo Seon Kwon, Jin Ha Hwang, Hwa Sung Shin*

Department of Biological Engineering, Inha University, Incheon, 402-751, Korea

+ Equally contributed

*** Corresponding author.**

Hwa Sung Shin, hsshin@inha.ac.kr, Tel: 82-32-860-9221, Fax: 82-32-872-4046

This file includes :
1 table and 7 figures which are supporting our research.

Gene	Forward (5'→3')	Reverse (3'→5')
MCP-1	GCTTGGTGGTTTGCTACGAC	ATGGGCTCCCTCTCATCAGT
TNF-α	TGATCCCAATGAGTCGGCTG	TGGACCCATTCCTTATTGGGG

Table S1. mRNA sequences that were designed for the analysis of inflammation of nanodrug treated rat *in-vivo* skin.

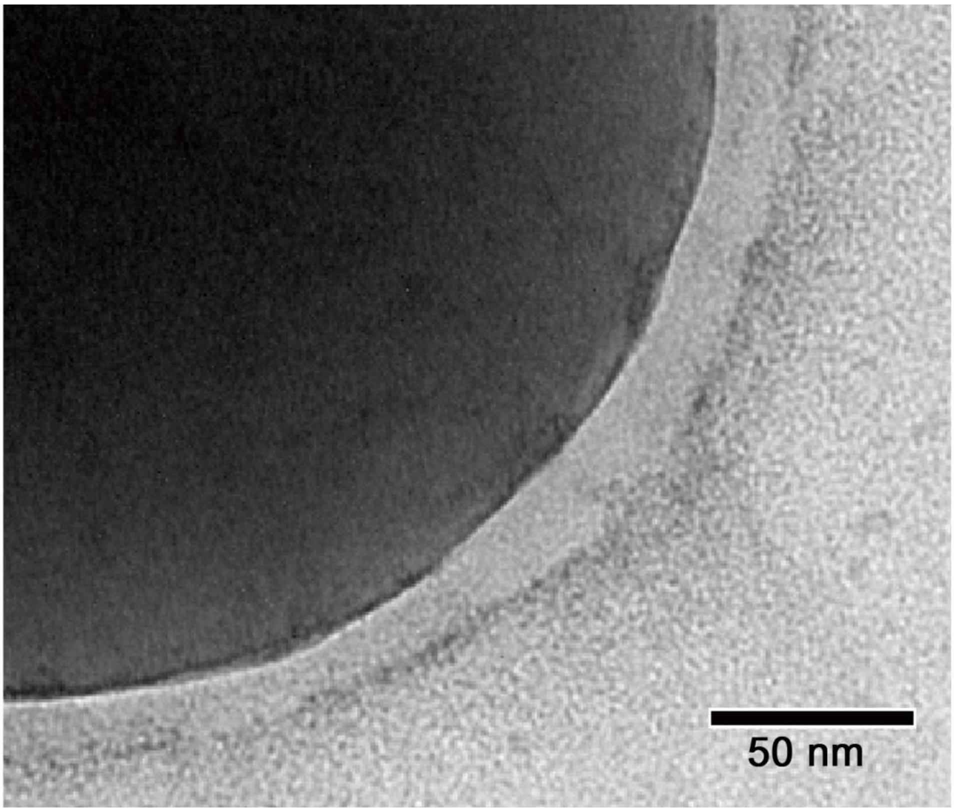

Figure S1. The TEM image expanding the boundary of PLGA nanoparticle and chitosan coating layer.

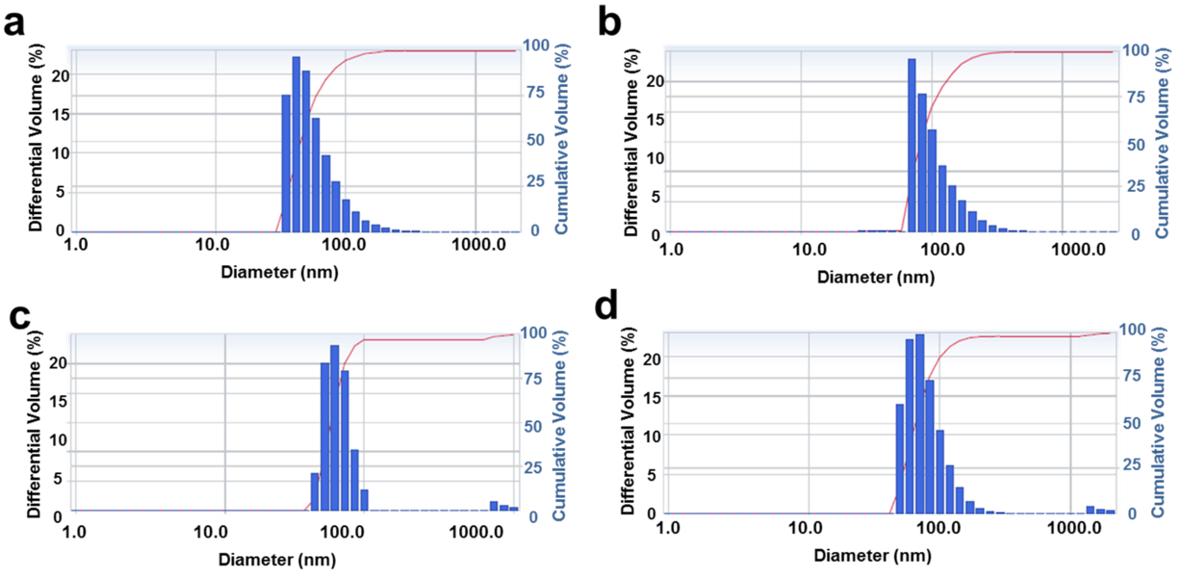

Figure S2. The diameter distributions of each nanoparticle using Zetasizer. (a) PLGA nanoparticle, (b) ceramide imbedded PLGA, (c) chitosan coated PLGA nanoparticles, and (d) Chi-PLGA/Cer.

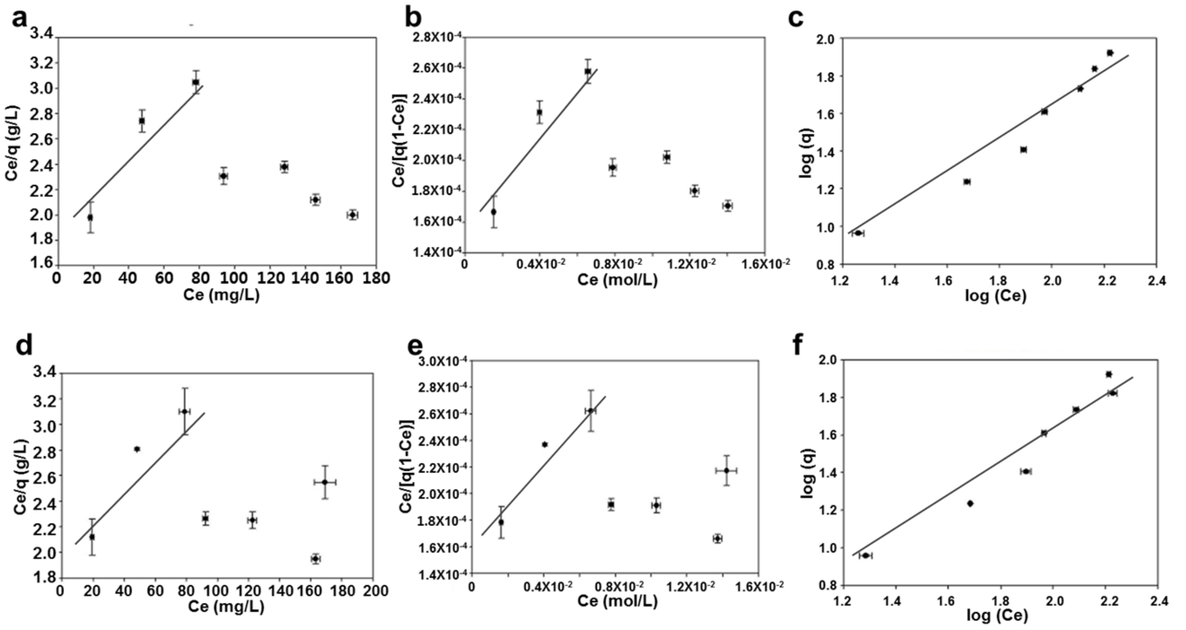

Figure S3. Linear graph derived Langmuir, BET and Freundlich isothermal model at 4℃. Comparison of the Chi-PLGA nanoparticles including no ceramide (a, b and c) and ceramide (d, e, and f) (C_e: The concentration of chitosan remaining in the equilibrium (mg/g in a, d; mol/L in b, c, e and f), q: The amount of the absorbed chitosan per unit weight of PLGA nanoparticle. (mg/g in a, c, d and f; mol/g in b, e)). The value was pressured as the means ± S.D. (n = 3).

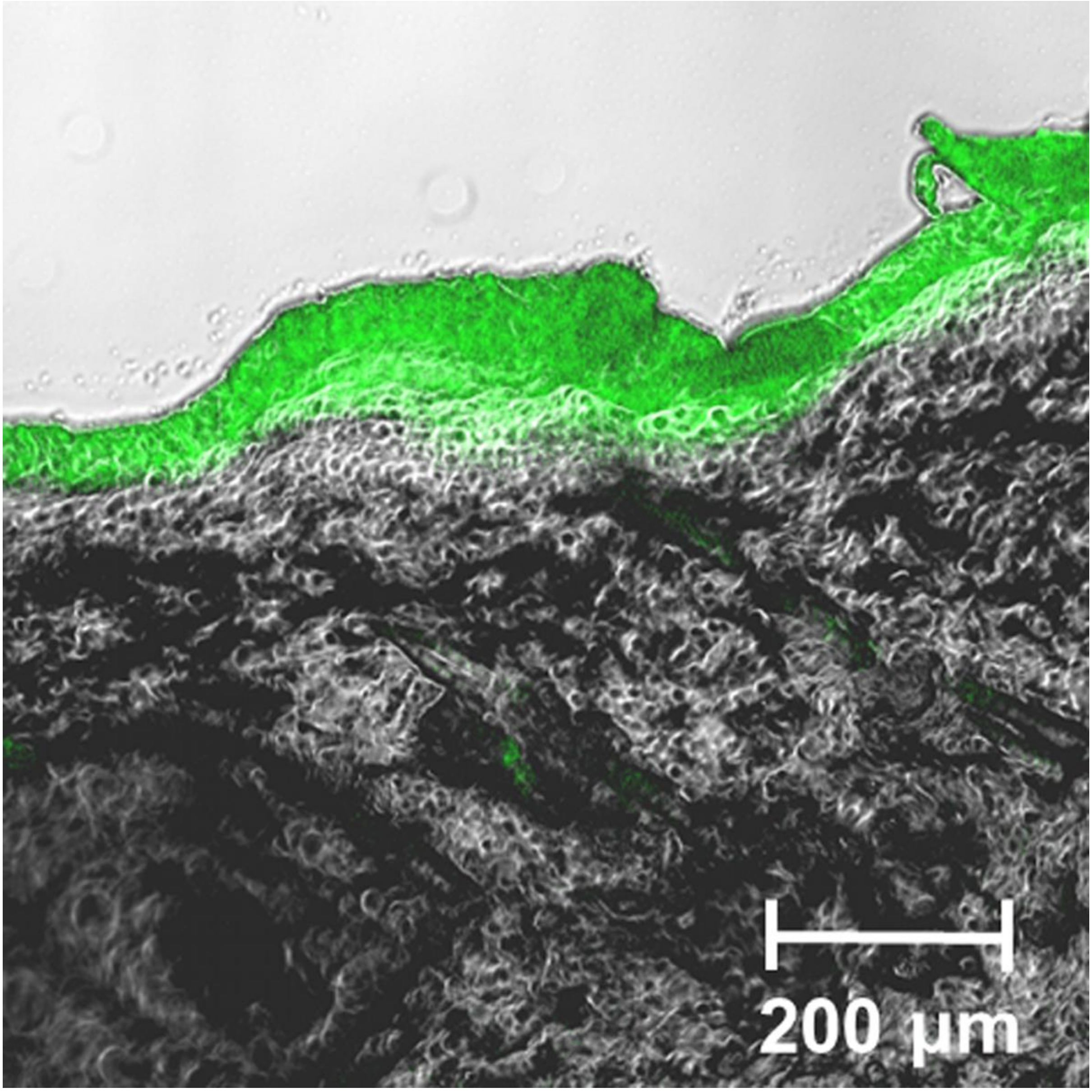

Figure S4. The fluorescent image of PLGA nanoparticles with FITC-labeled chitosan coating penetrated into stratum corneum of *ex-vivo*.

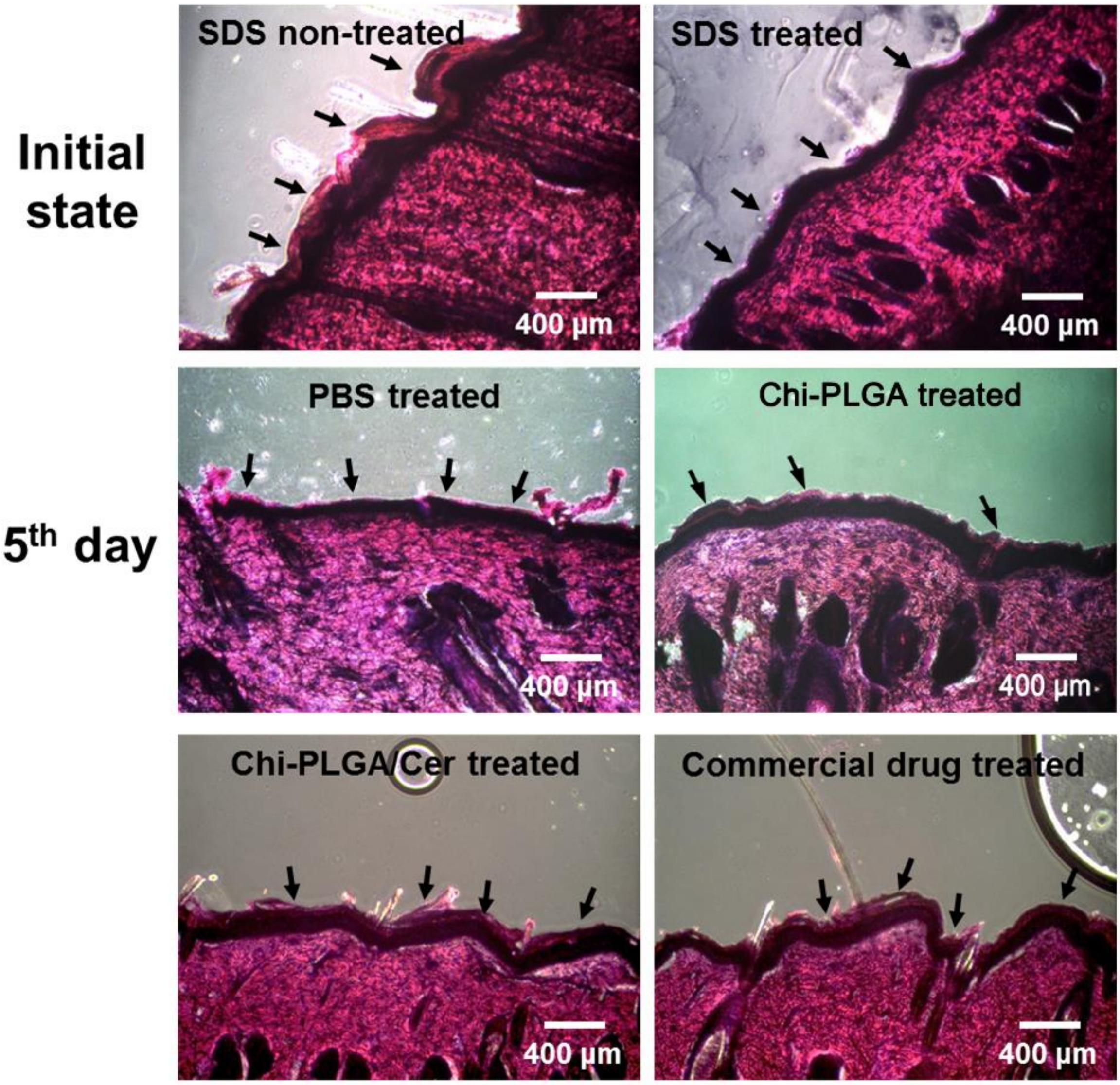

Figure S5. *In-vivo* assay was carried out to evaluate Chi-PLGA/Cer efficacy. Cross section images of PBS, Chi-PLGA/Cer and Commercial drug treated samples at initial state and 5th day.

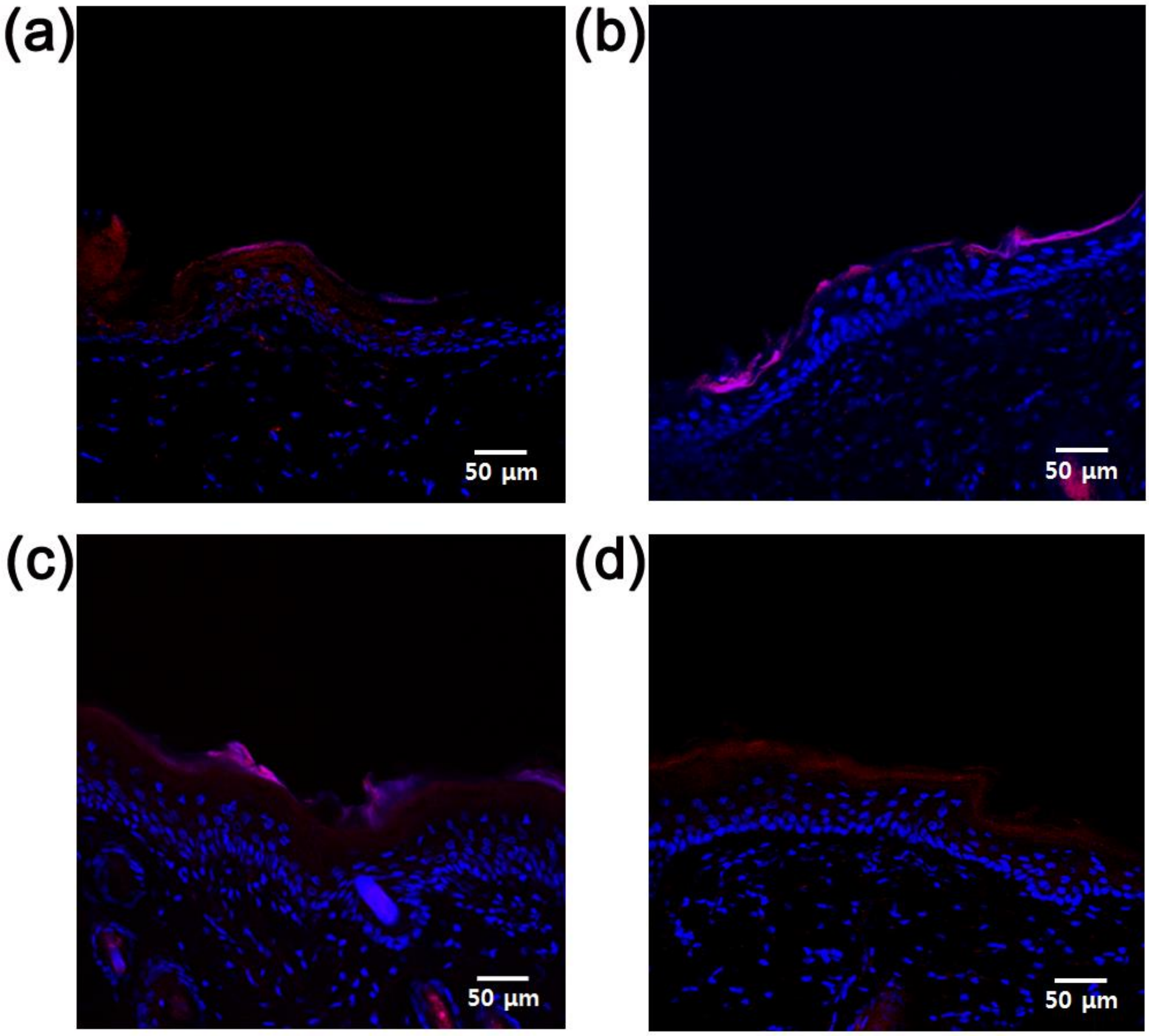

Figure S6. Immunostaining of filaggrin was conducted on the cross sections of rat skin tissues. The rat skin was secured after 7 day from sample treatment. (a) PBS treated, (b) Chi-PLGA treated, (c) Chi-PLGA/Cer treated and (d) Novason cream.

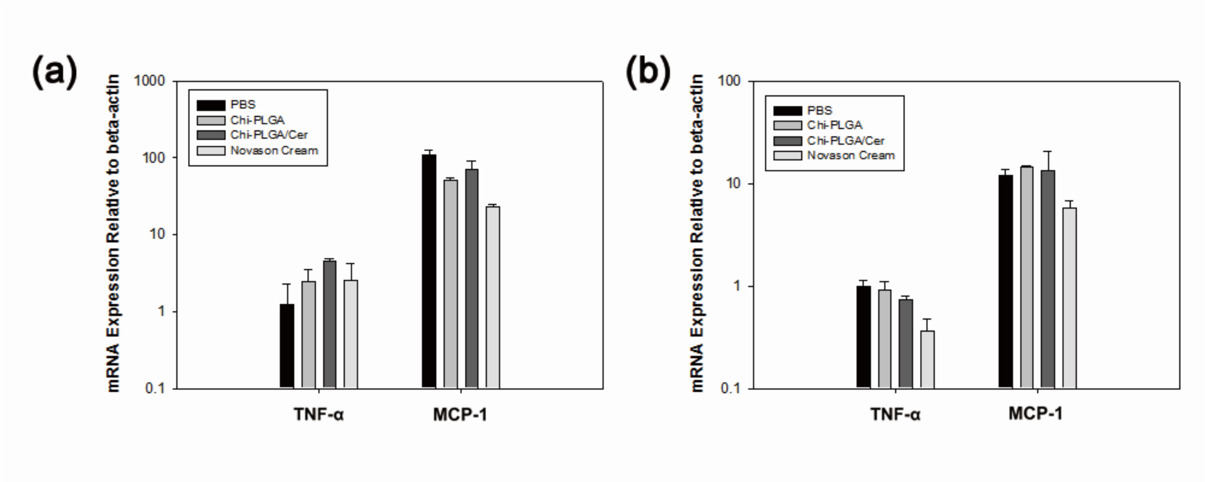

Figure S7. Inflammatory analysis by mRNA expression of the markers (MCP-1 and TNF-α) at (a) day 5 and (b) day 7 after sample treatment.
